# Persistent Organic Pollutants and Type 2 Diabetes: A Prospective Analysis in the Nurses’ Health Study and Meta-analysis

**DOI:** 10.1289/ehp.1205248

**Published:** 2012-11-05

**Authors:** Hongyu Wu, Kimberly A. Bertrand, Anna L. Choi, Frank B. Hu, Francine Laden, Philippe Grandjean, Qi Sun

**Affiliations:** 1 Department of Nutrition, and; 2 Department of Epidemiology, Harvard School of Public Health, Boston, Massachusetts, USA; 3 Channing Division of Network Medicine, Department of Medicine, Brigham and Women’s Hospital and Harvard Medical School, Boston, Massachusetts, USA; 4 Department of Environmental Health, Harvard School of Public Health, Boston, Massachusetts, USA; 5 Institute of Public Health, University of Southern Denmark, Odense, Denmark

**Keywords:** DDE, DDT, dichlorodiphenyldichloroethylene, dichlorodiphenyltrichloroethane, HCB, hexachlorobenzene, PCB, persistent organic pollutant, polychlorinated biphenyl, POP, type 2 diabetes

## Abstract

Background: Prospective data regarding persistent organic pollutants (POPs) and risk of type 2 diabetes (T2D) are limited, and the results for individual POPs are not entirely consistent across studies.

Objectives: We prospectively examined plasma POP concentrations in relation to incident T2D and summarized existing evidence in a meta-analysis.

Methods: Plasma polychlorinated biphenyls (PCBs), dichlorodiphenyltrichloroethane (DDT), dichlorodiphenyldichloroethylene (DDE), and hexachlorobenzene (HCB) concentrations were measured in 1,095 women who were free of diabetes at blood draw in 1989–1990 and participated in two case–control studies in the Nurses’ Health Study. We identified 48 incident T2D cases through 30 June 2008. We conducted a literature search in PubMed and EMBASE through December 2011 to identify prospective studies on POPs in relation to diabetes. We used a fixed-effects model to summarize results.

Results: After multivariable adjustment, plasma HCB concentration was positively associated with incident T2D [pooled odds ratio (OR) 3.59 (95% CI: 1.49, 8.64, *p*_trend_ = 0.003) comparing extreme tertiles]. Other POPs were not significantly associated with diabetes. After pooling our results with those of six published prospective studies that included 842 diabetes cases in total, we found that HCB and total PCBs both were associated with diabetes: the pooled ORs were 2.00 (95% CI: 1.13, 3.53; *I*^2^ = 21.4%, *p*_heterogeneity_ = 0.28) and 1.70 (95% CI: 1.28, 2.27; *I*^2^ = 16.3%, *p*_heterogeneity_ = 0.30) for HCB and total PCBs, respectively.

Conclusions: These findings support an association between POP exposure and the risk of T2D.

The prevalence of type 2 diabetes (T2D) is increasing rapidly worldwide, and this disease has posed an enormous public health challenge. It is widely considered that complex interactions between genetic and environmental factors may underlie the etiology of diabetes (Hu 2011). Growing evidence has recently linked risk of T2D with some environmental pollutants, such as persistent organic pollutants (POPs) (Everett et al. 2010).

POPs are a variety of synthetic compounds that can accumulate in human adipose tissue and are characterized by slow degradation (Milbrath et al. 2009). Common types of POPs include polychlorinated biphenyls (PCBs) and organochlorine pesticides such as dichlorodiphenyltrichloroethane (DDT) and its major metabolite, dichlorodiphenyldichloroethylene (DDE), and hexachlorobenzene (HCB). Contaminated fish, meat, and dairy food products are the primary sources of exposure in the general population (Milbrath et al. 2009). In addition, inhalation from indoor air and ingestion of dust have been suggested to be other important sources of PCB exposure (Harrad et al. 2009). Although the use of PCBs and DDT was banned decades ago, serum concentrations of these pollutants are still detectable in most of the U.S. population (Lee et al. 2007a). Animal studies have suggested that exposure to POPs may induce abdominal obesity, impair insulin sensitivity (Ruzzin et al. 2010), and reduce glucose uptake (Enan and Matsumura 1994). Several cross-sectional studies have reported that certain POPs were significantly associated with T2D (Airaksinen et al. 2011; Codru et al. 2007; Lee et al. 2006; Philibert et al. 2009; Rignell-Hydbom et al. 2007; Rylander et al. 2005). Overall, limited prospective data also suggest that exposure to POPs may increase the risk of T2D (Lee et al. 2010, 2011; Rignell-Hydbom et al. 2009; Turyk et al. 2009; Vasiliu et al. 2006; Wang et al. 2008); however, associations of individual POPs were not entirely consistent across the studies. For example, total PCBs were associated with risk of diabetes in some (Lee et al. 2011; Vasiliu et al. 2006), but not all studies (Turyk et al. 2009; Wang et al. 2008). Moreover, evidence for DDT/DDE (Audouze and Grandjean 2011; Lee et al. 2010) and HCB (Lee et al. 2010, 2011) is sparse. Small sample sizes, different exposure distributions, and other characteristics of study populations may potentially explain the inconsistency.

In the present study, we aimed to prospectively evaluate plasma concentrations of PCB congeners, DDT, DDE, and HCB in relation to risk of T2D using existing data from the Nurses’ Health Study (NHS). We also conducted a meta-analysis to summarize existing prospective evidence on relevant associations.

## Methods

*Study population.* The NHS is an ongoing prospective cohort of 121,700 female registered nurses who were 30 to 55 years old at study inception in 1976 when each nurse completed a mailed questionnaire about her medical history and lifestyle (Colditz et al. 1997). A total of 32,826 women provided blood samples in 1989 and 1990. Among these participants, multiple nested case–control studies were conducted to evaluate biomarkers and disease risk using the same design: For each disease outcome, incident cases were identified/confirmed among disease-free participants and matched controls were randomly selected using risk-set sampling. Participants for the present analyses were initially selected for two independent nested case–control studies: a non-Hodgkin lymphoma (NHL) study (Laden et al. 2010) and a breast cancer study (Laden et al. 2001). A total of 145 NHL cases (diagnosed before 2004) and 2 controls per case (*n* = 290) in the NHL study (Laden et al. 2010), and 381 pairs of breast cancer cases (diagnosed before 1994) and controls in the breast cancer study (Laden et al. 2001) were included. Measurements of plasma POPs and lipid concentrations before cancer diagnosis were available for 435 nurses from the NHL study and 744 nurses from the breast cancer study. These participants constituted the study population for the present analysis.

The study protocol was approved by the institutional review board of the Brigham and Women’s Hospital and the Human Subjects Committee Review Board of Harvard School of Public Health. All participants provided written informed consent.

*Ascertainment of T2D.* The study outcome in the present investigation was incident T2D diagnosed between the baseline blood draw in 1989–1990 and 30 June 2008. We sent a validated supplementary questionnaire (Manson et al. 1991) to participants who reported having diabetes in follow-up questionnaires to confirm the diagnosis of diabetes. In this supplementary questionnaire, we collected information on symptoms, diagnostic tests, and treatment. Over the years, the response rate to this supplementary questionnaire has approached 100%. For self-reported cases before 1998, we used the National Diabetes Data Group criteria (National Diabetes Data Group 1979) to confirm diagnosis of T2D. Since 1998, we have applied the American Diabetes Association criteria (American Diabetes Association 1997) to confirm the cases. The validity of the supplementary questionnaire for confirming the diagnosis of diabetes has been described previously (Manson et al. 1991). Of a random sample of 62 women reporting T2D in the supplementary questionnaire, the diagnosis was confirmed in 61 (98%) of them after their medical records were reviewed by an endocrinologist blinded to the supplementary questionnaire information (Manson et al. 1991).

*Exclusions.* We examined the diabetes diagnosis status among the 1,179 participants from the NHL and breast cancer studies. We excluded 81 participants who developed diabetes before blood draw. In addition, we excluded 3 participants who had missing POP data. After these exclusions, a total of 1,095 participants who were free of diabetes at blood collection were included in the analysis. Of these participants, we identified 48 incident T2D cases through June 2008. The remaining 1,047 nondiabetic participants served as population controls.

*Laboratory analyses of POPs.* The methods for measuring POP concentrations were described in detail in previous publications (Laden et al. 2001, 2010). In the present study, we focused on the four most abundant PCB congeners (118, 138, 153, and 180), *p,p*´-DDT, *p,p*´-DDE, and HCB. A total of 18 minor PCB congeners in the breast cancer study and 52 minor PCB congeners in the NHL study were measured as well, and their original concentrations were summed with the concentrations of the four major PCBs to calculate the total PCB concentration. Total POP concentrations were calculated by summing concentrations of total PCBs, DDT, DDE, and HCB. We also examined the associations for total PCBs and total POPs in each study.

Of note, there were some important differences in laboratory methods between the two contributing studies. For the breast cancer study, laboratory assays were performed at the Mount Sinai School of Medicine (New York, NY, USA) in 1994–1997 by single-column gas chromatography with electron capture detection (Laden et al. 2001). The limits of detection (LODs) were < 1 ng/mL for HCB, DDT, DDE, and PCBs. The detection rate was 95.2% for HCB, 98.3% for DDT, 99.8% for DDE, and > 99.4% for PCB congeners 118, 138, 153, and 180. The median coefficients of variation (CVs) in the breast cancer study were 5.0% for DDE, 12.0% for total PCBs, and 8.1–12.4% for the four main PCB congeners (Laden et al. 2001). In contrast, for the NHL study, laboratory assays were performed at the Harvard School of Public Health in 2004–2005 using dual capillary column gas chromatography to separate interfering peaks (Bertrand et al. 2010; Laden et al. 2010). The LODs ranged from 0.007 ng/mL for HCB to 0.039 ng/mL for PCB-180. The detection rate was 100% for HCB and DDE, 96.5% for DDT, and > 99.4% for the four major PCBs. Any value below LOD for a given pollutant was set to be the detection limit of that pollutant to preserve statistical power. The median CVs of measurement in the NHL study were < 7.5% for all POPs of interest.

We found reasonable correlations between POP concentrations in 30 samples that were assayed using both methods. The Spearman correlation coefficients were 0.53 for PCB-118, 0.60 for PCB-138, 0.75 for PCB-153, 0.77 for PCB-180, 0.76 for HCB, 0.92 for DDE (all *p*-values < 0.01), and 0.10 for DDT (*p* = 0.59). The two data sets were analyzed separately, as described below.

*Assessment of covariates.* Information about current body weight, lifestyle factors, and family history of diabetes was derived from the 1990 follow-up questionnaire (Colditz et al. 1997). Body mass index (BMI) was calculated as weight in kilograms divided by height in meters squared. Physical activity was expressed as metabolic equivalent task (MET) in hours per week. The validity of the self-reported body weight and physical activity levels has been described previously (Rimm et al. 1990; Wolf et al. 1994).

*Statistical analyses.* Because POPs are highly lipophilic and, therefore, predominantly carried by blood lipids, lipid-standardized POP concentrations [nanograms per gram of plasma total lipids derived using the Phillips formula (Phillips et al. 1989)] were used in the present analysis to minimize the impact of blood lipids on the associations of interest. We used logistic regression to estimate odds ratios (ORs) and 95% CIs of incident T2D risk by tertiles of POP concentrations that were defined separately for each study. In multivariable analysis, we adjusted for potential confounders, including age (years), smoking status (never, current smoker, past smoker), alcohol intake (0, 0.1–10, and > 10 g/day), physical activity (MET-hr/week), family history of diabetes (yes/no), and BMI at baseline, as well as cancer case–control status. To test for linear trend, we modeled the median concentrations of POP tertiles as a continuous variable. Natural log transformation of POP concentrations was applied to model linear associations between POP exposures and diabetes risk. Because of the apparent between-assay differences in POP assay methodology, we performed the above-mentioned analyses within each study separately and then pooled results using a fixed-effects model. To derive pooled *p*-values for trend, we pooled regression coefficients for the median concentrations of POP tertiles using a fixed-effects model and then estimated *p*-values for the pooled regression coefficients.

*Sensitivity analysis.* Because POP exposures were associated with elevated triglyceride concentrations (Lee et al. 2007b) and dyslipidemia is associated with T2D status (Mooradian 2009), the use of lipid-standardized POP concentrations may cause bias (Schisterman et al. 2005). Therefore, we also examined plasma weight-adjusted POP concentrations in relation to risk of T2D and controlled for plasma total cholesterol and triglycerides as covariates in multivariable models. Because there were no T2D cases in the lowest HCB tertile, when analyzing data for HCB, we categorized the study population using the following cutoff points: ≤ median, median to 75th percentile, and ≥ 75th percentile. In another sensitivity analysis, we used a nonparametric approach (Rosner and Glynn 2007) to derive a standardized score for each POP within each study to account for the differences in POP assays between studies. Briefly, within each data set, we transformed the POP concentrations to a probit scale to normalize the distribution, and then ranked the data to generate study-specific tertiles. We then pooled individual-level data from both studies and repeated the analysis. In addition, we conducted two separate sensitivity analyses to evaluate the possible impact of cancer treatment on the relationship. In the first analysis, we restricted our analysis to participants who did not develop cancer by the end of follow-up. In the second analysis, we excluded diabetes cases who reported occurrence of cancer prior to diabetes diagnosis (*n* = 5). To examine whether the associations could be due to reverse causation bias, in a further sensitivity analysis we excluded diabetes cases reported ≤ 2 years after blood sample collection in 1989–1990.

*Statistical analyses were performed using SAS version 9.1 (SAS Institute Inc., Cary, NC, USA).* All reported *p*-values are two-sided, and α = 0.05 was used as the significance level.

**Figure 1:**
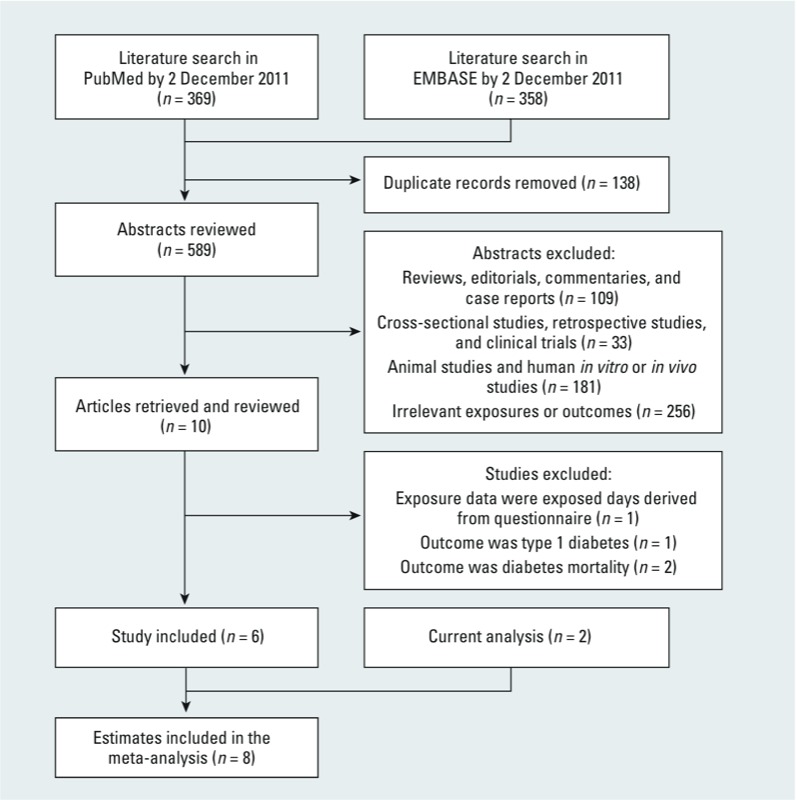
Literature search and study selection.

*Meta-analysis.* Study selection. We searched the National Institutes of Health U.S. National Library of Medicine’s PubMed (http://www.ncbi.nlm.nih.gov/pubmed/) and Elsevier’s EMBASE (http://www.embase.com/) databases for articles regarding POP exposures and diabetes risk that were published through 2 December 2011. [For a list of the search terms used, see Supplemental Material, p. 2 (http://dx.doi.org/10.1289/ehp.1205248).] We applied the following study inclusion criteria: *a*) a prospective study design, and *b*) that point estimates of relative risk (RR) of diabetes with 95% CI or SEs were available or could be derived. We excluded animal studies, clinical trials, cross-sectional studies, reviews, commentaries, letters, and studies that examined irrelevant exposures or outcomes. Two investigators (H.W. and K.A.B.) independently screened all studies by title or abstract, and then by a full text evaluation. Any discrepancy between the two authors was solved by discussion with the senior investigator (Q.S.). Of 589 unique publications identified in the literature search, we identified six prospective studies (Lee et al. 2010, 2011; Rignell-Hydbom et al. 2009; Turyk et al. 2009; Vasiliu et al. 2006; Wang et al. 2008) that explicitly evaluated the association between circulating POP concentrations and incident T2D (Figure 1).

*Data extraction.* We extracted the following information from each study: study characteristics (study name, authors, publication year, study design, duration of follow-up, and number of participants and incident cases), participant characteristics (age and sex), exposure (POP concentrations for each category) and measurement method of POPs, outcome ascertainment, and analysis strategy (comparison categories, risk estimates for the comparison categories, and covariates included in the fully adjusted models). Data extraction was conducted independently by two investigators (H.W. and K.A.B.), and any discrepancy was again solved by discussion with Q.S.

*Statistical analyses.* Meta-analyses were performed using Stata version 10.0 (StataCorp, College Station, TX, USA). We used a fixed-effects model to summarize study estimates comparing extreme categories (highest category vs. reference category) of POPs in each individual study. In this analysis, we used logarithms of RRs and corresponding SEs that were derived from the 95% CIs in each individual study. We also used a random-effects model to pool RRs across studies. Heterogeneity among the results of these studies was evaluated using the *I*^2^ statistic. We further used the meta-regression approach (Stata METAREG command) to evaluate whether the associations of POPs were influenced by some study characteristics such as age, sex, demographic variables, baseline POP concentrations, diabetes diagnosis strategies, duration of follow-up, and whether blood lipid was adjusted for in the final model. We also conducted a dose–response meta-analysis by using the generalized least-squares method for trend estimation of summarized dose–response data (Stata GLST command) (Greenland and Longnecker 1992). To evaluate a potential nonlinear dose–response relationship, we fitted two models: a restricted cubic spline regression model (Stata RC_SPLINE command) with four knots to create spline variables, and a linear regression model. We then examined the significance of nonlinear terms using the likelihood ratio test based on the statistics derived from these two models (Hu et al. 2012). To be consistent with previous studies, in this dose–response meta-analysis we used estimates that were based on plasma weight–adjusted POP concentrations. In addition, we excluded the Yucheng cohort (Wang et al. 2008) from the dose–response analysis because it included subjects who were poisoned by exposure to high levels of PCBs and polychlorinated dibenzofurans (PCDFs) in contaminated cooking oil (Guo et al. 1997).

## Results

*NHS.* Table 1 shows the baseline characteristics of participants in the two nested case–control studies. As expected, T2D patients had a significantly higher BMI at baseline and were more likely to have a family history of diabetes than were nondiabetic participants in both studies. We did not observe statistically significant differences in plasma POP concentrations between diabetes cases and nondiabetic participants in either study. The baseline characteristics of participants by cancer case–control status are shown in Supplemental Material, Table S1 (http://dx.doi.org/10.1289/ehp.1205248). The incident rate of T2D (cases/1,000 person-years) was significantly higher among cancer controls than cancer cases [3.63 vs. 0.57, *p* < 0.001 in the breast cancer study and 4.52 vs. 0.99, *p* = 0.005 in the NHL study (see Supplemental Material, Table S1)], probably because cancer cases had higher mortality than controls (see Supplemental Material, Table S1).

**Table 1:** Characteristics of NHS study participants at baseline in 1990.

Variable	Breast cancer study		NHL study	
	Diabetic (n = 24)	Nondiabetic (n = 649)	Diabetic (n = 24)	Nondiabetic (n = 398)
Age (years)	58.0 ± 6.3	58.6 ± 6.8	59.6 ± 5.7	58.7 ± 6.5
BMI (kg/m2)	30.1 ± 6.4	24.7 ± 4.1	27.6 ± 5.6	24.9 ± 4.1
Smoking status [n (%)]
Never smoked	11 (45.8)	297 (45.8)	13 (54.2)	178 (44.7)
Past smoker	11 (45.8)	276 (42.5)	6 (25.0)	177 (44.5)
Current smoker	2 (8.4)	76 (11.7)	5 (20.8)	43 (10.8)
Alcohol drinking, g/day [n (%)]
0	8 (33.3)	113 (17.4)	8 (33.3)	104 (26.1)
0.1–10	13 (54.2)	361 (55.6)	15 (62.5)	208 (52.3)
> 10	3 (12.5)	175 (27.0)	1 (4.2)	86 (21.6)
Physical activity (MET-hr/week)	18.3 ± 20.6	17.6 ± 16.4	15.0 ± 12.9	18.7 ± 34.3
Family history of diabetes [n (%)]	9 (37.5)	153 (23.6)	14 (58.3)	108 (27.1)
PCB-118 (ng/g lipid)a	69.1 (54.8–107.6)	65.7 (46.6–85.6)	61.0 (43.3–79.0)	47.8 (34.2–73.4)
PCB-138 (ng/g lipid)a	95.9 (67.5–132.0)	94.4 (69.7–131.1)	58.3 (50.6–89.1)	64.1 (44.9–87.0)
PCB-153 (ng/g lipid)a	116.7 (79.5–161.7)	104.0 (80.8–141.6)	98.3 (82.0–123.7)	107.3 (78.6–139.0)
PCB-180 (ng/g lipid)a	71.3 (51.5–93.6)	74.6 (56.6–97.6)	65.7 (54.5–83.6)	71.8 (55.2–90.1)
∑PCBs (118,138,153,180) (ng/g lipid)a	365.2 (249.5–506.8)	346.0 (265.9–449.6)	291.8 (249.4–357.0)	300.7 (219.1–384.7)
Total PCBs (ng/g lipid)b	721.0 (604.5–1031.7)	742.8 (572.1–949.0)	628.2 (494.8–729.5)	621.7 (465.8–783.3)
p,p´-DDT (ng/g lipid)a	51.3 (37.6–114.6)	53.5 (32.5–94.9)	54.8 (31.4–76.2)	43.5 (28.1–67.3)
p,p´-DDE (ng/g lipid)a	826.5 (490.8–1435.2)	773.0 (453.3–1215.6)	1206.5 (817.9–1936.5)	973.8 (569.9–1717.8)
HCB (ng/g lipid)a	34.1 (27.0–43.3)	29.5 (22.0–39.0)	41.6 (33.3–47.7)	36.7 (30.0–45.5)
Total plasma cholesterol (mg/dL)	235.3 (193.8–265.1)	227.3 (200.2–253.0)	237.0 (182.5–282.0)	219.0 (190.0–243.0)
Plasma triglycerides (mg/dL)	184.5 (115.0–248.0)	103.0 (71.0–154.0)	194.5 (145.0–242.5)	110.5 (76.0–161.0)
Data are means ± SDs or medians (interquartile ranges) for continuous variables and n (%) for categorical variables. aAll POPs were adjusted for total plasma lipids derived using the Phillips formula: total plasma lipid = (2.27 × total plasma cholesterol) + plasma triglycerides + 0.623. bTotal PCBs were summed values of 22 PCB congeners in the breast cancer study and 56 PCB congeners in the NHL study.					

We examined age-adjusted Spearman correlations among plasma POPs, as well as between baseline BMI and POPs [see Supplemental Material, Table S2 (http://dx.doi.org/10.1289/ehp.1205248)]. The four major PCB congeners were highly correlated with each other in both studies, and all of the pairwise correlation coefficients were > 0.53. Positive associations were also observed among DDT, DDE, and HCB, although the correlations were somewhat weaker in the breast cancer study than in the NHL study. In general, BMI at baseline was inversely correlated with PCBs in both studies, except PCB-118 in the NHL study for which the correlation was positive (*r_S_* = 0.17). The correlations between BMI and DDT, DDE, and HCB were very weak (|*r_S_*| < 0.10), with the exception of the correlation between BMI and DDT in the NHL study (*r_S_* = 0.20).

Table 2 shows the association between plasma POP concentrations and diabetes in each study. Overall, patterns of associations were similar between the two studies. Of the POPs evaluated, HCB showed the strongest association with T2D. In the multivariable analysis adjusted for age, BMI, and other covariates (model 1), the ORs of T2D comparing extreme tertiles were 3.73 (95% CI: 1.05, 13.3; *p*_trend_ = 0.04) in the breast cancer study, and 3.46 (95% CI: 1.02, 11.7; *p*_trend_ = 0.03) in the NHL study. The pooled OR based on a fixed-effects model was 3.59 (95% CI: 1.49, 8.64; *p*_trend_ = 0.003) comparing extreme tertiles (Table 3, model 1). Further adjustment of NHL and breast cancer case–control status (Tables 2 and 3, model 2) did not materially change the study-specific or pooled results. When we modeled the association between natural log-transformed plasma POP concentrations and T2D risk, we estimated that per SD increment of HCB was associated with a pooled OR of 2.38 (95% CI: 1.03, 5.48, *p* = 0.04) in multivariable adjustment model [see Supplemental Material, Table S3, model 1 (http://dx.doi.org/10.1289/ehp.1205248)]. In pooled analysis, total PCBs, total concentrations of the four major PCB congeners (ΣPCBs), and other individual POPs, except PCB-138, were also associated with a nonsignificantly increased T2D risk. Total POPs, which were the sum of total PCBs, DDT, DDE, and HCB, showed nonsignificantly positive association with risk of T2D: The multivariable-adjusted (Table 2, model 1) ORs were 1.95 (95% CI: 0.62, 6.16; *p*_trend_ = 0.27) and 1.50 (95% CI: 0.49, 4.64; *p*_trend_ = 0.50) comparing extreme tertiles in the breast cancer study and the NHL study, respectively. The pooled OR was 1.71 (95% CI: 0.76, 3.82; *p*_trend_ = 0.22) comparing the highest to the lowest tertile (Table 3, model 1).

**Table 2:** Adjusted ORs (95% CIs) of incident diabetes according to the tertiles of lipid-standardized plasma POP concentrations (ng/g lipids) in 1990, the NHS.

POP	Breast cancer study				NHL study			
	Tertile 1	Tertile 2	Tertile 3	ptrend	Tertile 1	Tertile 2	Tertile 3	ptrend
HCB
Median	19.5	29.8	44.2		27.5	37.0	51.5
Case/control	4/220	9/216	11/213		5/135	7/134	12/129
Model 1a	1.00	2.13 (0.60, 7.58)	3.73 (1.05, 13.3)	0.04	1.00	1.44 (0.40, 5.24)	3.46 (1.02, 11.7)	0.03
Model 2b	1.00	1.74 (0.47, 6.50)	2.76 (0.75, 10.1)	0.12	1.00	1.36 (0.36, 5.07)	3.52 (1.03, 12.1)	0.03
p,p´-DDE
Median	349.5	773.6	1535.3		424.8	989.6	2099.5
Case/control	6/218	9/216	9/215		5/135	9/132	10/131
Model 1a	1.00	1.47 (0.48, 4.49)	1.59 (0.50, 5.03)	0.47	1.00	1.73 (0.54, 5.50)	1.57 (0.49, 5.07)	0.58
Model 2b	1.00	1.13 (0.35, 3.64)	1.32 (0.41, 4.27)	0.64	1.00	1.65 (0.51, 5.38)	1.79 (0.54, 5.86)	0.41
p,p´-DDT
Median	26.9	53.1	120.9		23.7	43.7	83.3
Case/control	7/217	8/217	9/215		7/133	7/134	10/131
Model 1a	1.00	1.11 (0.38, 3.25)	1.11 (0.38, 3.27)	0.87	1.00	0.81 (0.26, 2.49)	1.01 (0.34, 3.02)	0.90
Model 2b	1.00	1.17 (0.39, 3.54)	1.08 (0.36, 3.22)	0.94	1.00	0.75 (0.24, 2.40)	1.01 (0.34, 3.07)	0.86
PCB-118
Median	41.0	65.7	101.4		29.0	48.8	87.9
Case/control	6/218	9/216	9/215		6/134	7/134	11/130
Model 1a	1.00	1.50 (0.48, 4.63)	1.50 (0.48, 4.65)	0.53	1.00	1.18 (0.36, 3.92)	1.68 (0.51, 5.52)	0.37
Model 2b	1.00	1.34 (0.42, 4.27)	1.32 (0.42, 4.20)	0.68	1.00	1.16 (0.34, 3.93)	1.50 (0.45, 5.06)	0.49
PCB-138
Median	59.5	94.4	148.2		38.7	63.7	103.4
Case/control	9/215	6/219	9/215		6/134	11/130	7/134
Model 1a	1.00	0.74 (0.24, 2.26)	1.17 (0.42, 3.28)	0.70	1.00	1.40 (0.47, 4.16)	0.91 (0.28, 2.96)	0.76
Model 2b	1.00	0.69 (0.22, 2.16)	0.97 (0.33, 2.84)	0.96	1.00	1.40 (0.45, 4.32)	0.91 (0.27, 3.05)	0.75
PCB-153
Median	69.1	104.5	170.5		67.7	106.1	153.8
Case/control	8/216	6/219	10/214		7/133	11/130	6/135
Model 1a	1.00	0.87 (0.27, 2.80)	2.29 (0.77, 6.79)	0.09	1.00	1.22 (0.43, 3.50)	0.77 (0.23, 2.58)	0.64
Model 2b	1.00	0.85 (0.26, 2.78)	2.19 (0.72, 6.68)	0.12	1.00	1.26 (0.42, 3.78)	0.76 (0.22, 2.60)	0.61
PCB-180
Median	50.6	74.6	111.0		50.2	71.4	100.2
Case/control	8/216	9/216	7/217		9/131	10/131	5/136
Model 1a	1.00	1.75 (0.59, 5.16)	1.87 (0.57, 6.09)	0.32	1.00	1.11 (0.40, 3.09)	0.60 (0.17, 2.12)	0.42
Model 2b	1.00	1.66 (0.54, 5.08)	1.95 (0.57, 6.70)	0.31	1.00	1.13 (0.39, 3.23)	0.68 (0.19, 2.49)	0.55
∑PCBs (118,138,153,180)
Median	232.2	347.6	518.9		197.2	298.1	440.1
Case/control	10/214	4/221	10/214		5/135	13/128	6/135
Model 1a	1.00	0.49 (0.14, 1.67)	1.29 (0.47, 3.51)	0.53	1.00	1.91 (0.62, 5.87)	0.97 (0.27, 3.53)	0.76
Model 2b	1.00	0.40 (0.12, 1.41)	1.21 (0.43, 3.39)	0.59	1.00	2.26 (0.70, 7.27)	0.98 (0.26, 3.72)	0.72
Total PCBs
Median	521.9	742.6	1094.9		422.9	621.0	866.2
Case/control	8/216	7/218	9/215		7/133	10/131	7/134
Model 1a	1.00	1.08 (0.36, 3.25)	1.38 (0.47, 4.03)	0.54	1.00	1.10 (0.38, 3.22)	0.83 (0.25, 2.75)	0.74
Model 2b	1.00	1.21 (0.38, 3.80)	1.30 (0.43, 3.93)	0.65	1.00	1.25 (0.41, 3.80)	0.79 (0.23, 2.71)	0.65
Total POPs
Median	1054.9	1681.9	2702.5		1042.9	1779.2	2957.8
Case/control	8/216	7/218	9/215		5/135	13/128	6/135
Model 1a	1.00	1.57 (0.50, 4.96)	1.95 (0.62, 6.16)	0.27	1.00	1.32 (0.42, 4.12)	1.50 (0.49, 4.64)	0.50
Model 2b	1.00	1.31 (0.39, 4.37)	1.56 (0.48, 5.05)	0.47	1.00	1.19 (0.37, 3.87)	1.55 (0.49, 4.93)	0.45
ORs (95% CIs) were estimated using logistic regression. aModel 1: adjusted for age (years), smoking status (never/current smoker/past smoker), alcohol intake (0, 0.1–10, and > 10 g/day), physical activity (MET-hr/week), family history of diabetes (yes/no), and baseline BMI in 1990. bModel 2: further adjusted for cancer case–control status.									

**Table 3:** Pooled adjusted ORs (95% CIs) of incident diabetes according to the tertiles of lipid-standardized plasma POP concentrations (ng/g lipids), the NHS.

POP	Study-specific tertiles			ptrend
	1	2	3	
HCB
Case/control	9/355	16/350	23/342
Model 1a	1.00	1.76 (0.71, 4.34)	3.59 (1.49, 8.64)	0.003
Model 2b	1.00	1.54 (0.61, 3.90)	3.14 (1.28, 7.67)	0.003
p,p´-DDE
Case/control	11/353	18/348	19/346
Model 1a	1.00	1.59 (0.71, 3.55)	1.58 (0.69, 3.59)	0.39
Model 2b	1.00	1.36 (0.59, 3.13)	1.53 (0.66, 3.53)	0.31
p,p´-DDT
Case/control	14/350	15/351	19/346
Model 1a	1.00	0.95 (0.44, 2.07)	1.06 (0.49, 2.28)	0.84
Model 2b	1.00	0.95 (0.43, 2.11)	1.05 (0.48, 2.28)	0.90
PCB-118
Case/control	12/352	16/350	20/345
Model 1a	1.00	1.34 (0.59, 3.05)	1.58 (0.70, 3.59)	0.28
Model 2b	1.00	1.25 (0.54, 2.90)	1.41 (0.61, 3.25)	0.43
PCB-138
Case/control	15/349	17/349	16/349
Model 1a	1.00	1.02 (0.47, 2.24)	1.05 (0.48, 2.28)	0.89
Model 2b	1.00	0.99 (0.44, 2.20)	0.94 (0.42, 2.11)	0.89
PCB-153
Case/control	15/349	17/349	16/349
Model 1a	1.00	1.05 (0.48, 2.29)	1.41 (0.63, 3.15)	0.32
Model 2b	1.00	1.05 (0.47, 2.35)	1.36 (0.59, 3.10)	0.47
PCB-180
Case/control	17/347	19/347	12/353
Model 1a	1.00	1.38 (0.66, 2.89)	1.10 (0.46, 2.61)	0.76
Model 2b	1.00	1.35 (0.63, 2.91)	1.18 (0.48, 2.89)	0.71
∑PCBs (118,138,153,180)
Case/control	15/349	17/349	16/349
Model 1a	1.00	1.02 (0.45, 2.34)	1.16 (0.52, 2.55)	0.76
Model 2b	1.00	1.01 (0.43, 2.38)	1.12 (0.49, 2.53)	0.79
Total PCBs
Case/control	15/349	17/349	16/349
Model 1a	1.00	1.09 (0.51, 2.35)	1.10 (0.50, 2.45)	0.76
Model 2b	1.00	1.23 (0.55, 2.73)	1.04 (0.46, 2.38)	0.92
Total POPs
Case/control	15/349	17/349	16/349
Model 1a	1.00	1.43 (0.64, 3.23)	1.71 (0.76, 3.82)	0.22
Model 2b	1.00	1.25 (0.54, 2.90)	1.55 (0.68, 3.54)	0.30
Data were pooled ORs (95% CIs) of the estimates of the NHL and breast cancer studies, using a fixed-effects model. To derive pooled ptrend values, we pooled regression coefficients for the median concentrations of POP tertiles using a fixed-effects model and then estimated p-values for the pooled regression coefficients. aModel 1: adjusted for age (years), smoking status (never/current smoker/past smoker), alcohol intake (0, 0.1–10, and > 10 g/day), physical activity (MET-hr/week), family history of diabetes (yes/no), and baseline BMI in 1990. bModel 2: further adjusted for cancer case–control status.					

*Sensitivity analysis.* We observed similar associations when modeling plasma weight-adjusted POPs and adjusting for plasma total cholesterol and triglycerides as covariates in multivariable models [see Supplemental Material, Tables S4 and S5 (http://dx.doi.org/10.1289/ehp.1205248)]. HCB still showed the strongest association with risk of T2D. The pooled OR was 3.76 (95% CI: 1.50, 9.44; *p*_trend_ = 0.005) comparing extreme tertiles. Other POPs did not show significant association with diabetes based on this analysis. When we used a nonparametric approach to derive a standardized score to account for the between-study differences, we again found similar associations (see Supplemental Material, Table S6). For example, in comparison to women in the lowest HCB score tertile, women in the highest tertile had an OR of 3.79 (95% CI: 1.54, 9.34; *p*_trend_ = 0.003), and other POPs were not significantly associated with diabetes. We observed largely similar results among cancer-free participants based on pooled fixed-effects estimates (see Supplemental Material, Table S7). After excluding T2D cases that occurred ≤ 2 years after blood collection (*n* = 7) or T2D cases that had any prior cancer diagnosis (*n* = 5), multivariable-adjusted ORs for the highest versus lowest tertiles of HCB were 3.91 (95% CI: 1.46, 10.5) and 3.03 (95% CI: 1.26, 7.28), respectively. Estimates for other POPs were also comparable to those for the main analysis (data not shown).

*Meta-analysis.* The characteristics of the six published prospective studies that evaluated circulating POP concentrations in relation to incident diabetes are shown in Table 4. Most studies included both men and women, except one study (Rignell-Hydbom et al. 2009) that included women only. Three studies reported associations for men and women separately (Turyk et al. 2009; Vasiliu et al. 2006; Wang et al. 2008). The specific POPs investigated varied across these studies: Four studies examined total PCBs (Lee et al. 2011; Turyk et al. 2009; Vasiliu et al. 2006; Wang et al. 2008), four studies examined DDE (Lee et al. 2010, 2011; Rignell-Hydbom et al. 2009; Turyk et al. 2009), three studies evaluated PCB-118 and PCB-153 independently (Lee et al. 2010, 2011; Turyk et al. 2009), two studies assessed PCB-180 and HCB (Lee et al. 2010, 2011), and only one study examined PCB-138 (Lee et al. 2011) or DDT (Lee et al. 2010). Most of these studies used logistic regression to examine the association between POPs and incident diabetes (Lee et al. 2010, 2011; Rignell-Hydbom et al. 2009; Wang et al. 2008), except that Turyk et al. (2009) used Cox regression and Vasiliu et al. (2006) used Poisson regression. The number of incident diabetes cases ranged from 36 to 371 among these studies. In total, including our study, there were 842 diabetes cases.

**Table 4:** Characteristics of prospective studies regarding exposure levels of POPs in relation to incident diabetes.

Reference	Study participants	Exposure and assay method	Outcome and ascertainment	Comparison categories	RR (95% CI)	Covariates in the fully adjusted model
Prospective cohort studies	
Vasiliu et al. 2006	The Michigan polybrominated biphenyl (PBB) cohort (USA) Total n: 1,384 Female: 50.3% Age: ≥ 20 years Follow-up: 25 years	Serum PBBs and PCBs measured using gas chromatography	Incident diabetes cases: total n = 180 (89 men and 91 women); diabetes was identified by self-report	For serum PCBs: highest (> 10 ppb) vs. lowest (≤ 5 ppb) group for men and women	Men: 1.74 (0.91, 3.34) Women: 2.33 (1.25, 4.34)	Age, BMI, smoking, alcohol consumption at enrollment, and serum PBB concentrations
Wang et al. 2008	The Yucheng cohort (Taiwan, China) Total n: 378 Yucheng subjects poisoned through ingesting PCB-contaminated oil in 1978–1979 and 370 matched reference subjects with background-level exposures Female: 59.0% Age: > 30 years Follow-up: 24 years	Serum concentrations of a mixture of 33 PCBs measured using gas chromatography	Incident T2D cases: total n = 81 (44 men and 37 women); T2D was identified through self-report	Yucheng vs. reference group; mean ± SD of serum PCBs (ppb) were 73.3 ± 86.3 for male Yucheng participants, and 87.4 ± 151.0 for female Yucheng participants, and the mean of total PCBs in the reference group was 1.67.	Men: 1.0 (0.5, 1.9) Women: 2.1 (1.1, 4.5)	Age and BMI for women; age, BMI, cigarette smoking, and alcohol drinking for men
Lee et al. 2011	The Prospective Investigation of the Vasculature in Uppsala Seniors (PIVUS) study (Sweden) Total n: 725 Female: 51.7% Age: 70 years Follow-up: 5 years	Plasma concentrations of 19 POPs (14 PCBs, 3 organochlorine pesticides, 1 brominated diphenyl ether, and 1 dioxin) measured using high-resolution chromatography coupled with high-resolution mass spectrometry	Incident T2D cases: total n = 36; T2D was identified as a fasting blood glucose ≥ 6.2 mmol/L or self-report of use of insulin or oral hypoglycemic agents	Highest vs. lowest quintile of POP concentrations (pg/g wet weight); PCB-118: 309–1,637 vs. 25.0–125; PCB-138: 1,206–2,739 vs. 107–563; PCB-153: 1,957–4,672 vs. 117–1,007; PCB-180: 1,585–7,865 vs. 153–858; DDE: 4,040–23,271 vs. 11.0–902; HCB: 370–4,252 vs. 88.0–173; ΣPCBs: not available	3.6 (0.7, 18.8) for PCB-118; 3.2 (0.8, 13.2) for PCB-138; 1.7 (0.5, 6.2) for PCB-153; 4.8 (1.1, 20.9) for PCB-180; 2.1 (0.7, 6.3) for DDE; 2.1 (0.6, 7.1) for HCB; 7.5 (1.4, 38.8) for ΣPCBs	Sex, BMI, cigarette smoking, alcohol consumption, exercise, triglycerides, and total cholesterol
Turyk et al. 2009	The Great Lakes Consortium for the Health Assessment of Great Lakes Sport Fish Consumption (USA) Total n: 471 Female: 40.8% Age: mean = 52.2 and 47.9 years for diabetes and nondiabetes subjects, respectively Follow-up: 11 years	Serum concentrations of DDE, PCB-118, and ΣPCBs (sum of PCB congeners 74, 99, 118, 146, 180, 194, 201, 206, 132/153, 138/163, 170/190, 182/187, and 196/20) measured using gas chromatography	Incidence diabetes cases: total n = 36; diabetes was identified by self-report	Highest vs. lowest tertile of POP concentrations (ng/g wet weight); DDE: 5.4–49.2 vs. < LOD to 2.2; ΣPCBs: 4.3–29.8 vs. < LOD to 1.6; PCB-118: 0.3–4.6 vs. < LOD to 0.1	7.1 (1.6, 31.9) for DDE; 1.8 (0.6, 5.0) for ΣPCBs; 1.3 (0.5, 3.0) for PCB-118	Age, age squared, BMI, sex, serum lipids, smoking, alcohol use, all fish meals in the last year, and Great Lakes sport-caught fish meals in the last year
Case–control studies	
Rignell-Hydbom et al. 2009	The Women’s Health In the Lund Area cohort study (Sweden) Total n: 742 (371 cases and 371 controls) Female: 100% Age: 50–59 years Follow-up: 11 years	Serum concentrations of PCB-153 and DDE measured using high-resolution mass spectrometry	Incident T2D cases: total n = 371; T2D was identified by linkage with the Swedish inpatient and out-patient registers	Highest vs. the other three lower quartiles combined; PCB-153: > 1,790 pg/mL vs. ≤ 1,790 pg/mL; DDE: > 4,600 pg/mL vs. ≤ 4,600 pg/mL	0.99 (0.71, 1.4) for PCB-153; 1.1 (0.76, 1.5) for DDE	None
Lee et al. 2010	The Coronary Artery Risk Development in Young Adults (CARDIA) cohort study (USA); Total n: 180 (90 cases and 90 controls) Female: 46.6% Age: 18–30 years Follow-up: 18 years	Serum concentrations of 31 POPs (8 organochlorine pesticides, 22 PCBs, and 1 PBB congener) measured using gas chromatography isotope dilution high-resolution mass spectrometry	Incident T2D cases: total n = 90; diabetes was defined as ever having taken antidiabetic medications or ever having had fasting glucose ≥ 126 mg/dL at ≥ 2 examinations	Highest vs. lowest quartile of POP concentrations (pg/g wet weight); HCB: not available; DDE: > 5,731 vs. ≤ 2,153; DDT: not available; PCB-118: not available; PCB-153: > 466 vs. ≤ 204; PCB-180: not available	1.0 (0.4, 2.6) for HCB; 0.7 (0.2, 1.9) for DDE; 0.9 (0.3, 2.6) for DDT; 0.5 (0.2, 1.4) for PCB-118; 0.8 (0.2, 2.6) for PCB-153; 1.1 (0.3, 3.9) for PCB-180	Age, sex, race, BMI, triglycerides, and total cholesterol


**Figure 2:**
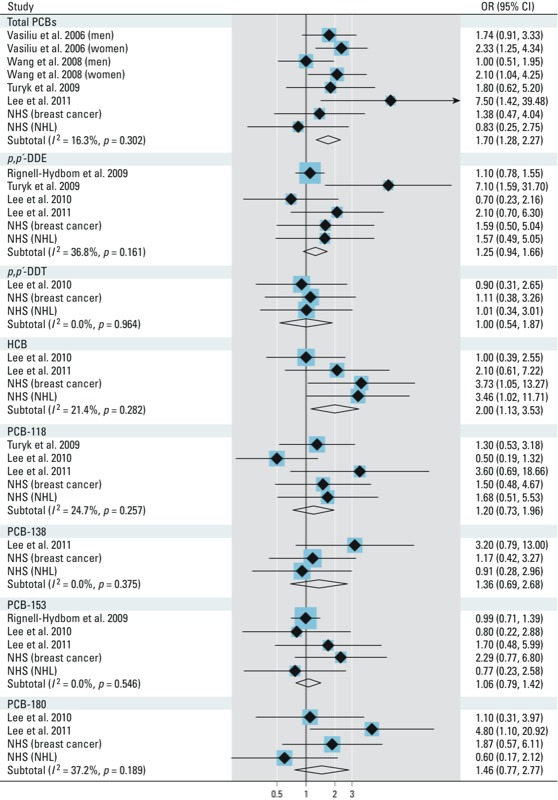
Pooled fixed-effects ORs (95% CIs) of incident diabetes comparing extreme categories (the highest vs. the lowest) of POP concentrations; *p-*values are *p*_heterogeneity_.

When all data were pooled using a fixed-effects model, high concentrations of total PCBs and HCB were significantly associated with risk of diabetes, and the test for heterogeneity was not significant (Figure 2). The pooled ORs of diabetes comparing high versus low concentrations were 1.70 (95% CI: 1.28, 2.27; *I*^2^ = 16.3%, *p*_heterogeneity_ = 0.30) for total PCBs and 2.00 (95% CI: 1.13, 3.53; *I*^2^ = 21.4%, *p*_heterogeneity_ = 0.28) for HCB. Of note, estimates for HCB based on data from two previous studies (Lee et al. 2010, 2011) and the NHL and breast cancer studies in the NHS. Most other POPs of interest showed positive associations, although none of these associations achieved statistical significance. For example, the pooled ORs of diabetes for high versus low concentrations were 1.25 (95% CI: 0.94, 1.66; *I*^2^ = 36.8%, *p*_heterogeneity_ = 0.16) for DDE, 1.20 (95% CI: 0.73, 1.96; *I*^2^ = 24.7%, *p*_heterogeneity_ = 0.26) for PCB-118, and 1.36 (95% CI: 0.69, 2.68; *I*^2^ = 0.0%, *p*_heterogeneity_ = 0.38) for PCB-138 (Figure 2). When we used a random-effects model to pool these data, we found similar results (data not shown).

Meta-regression analysis indicated that the association between total PCBs and HCB and diabetes risk was not dependent on age, sex, other demographic variables, baseline total PCB or HCB concentrations, diabetes diagnosis strategies, duration of follow-up, or whether blood lipids were adjusted for in the final model (all *p* > 0.05, data not shown). The pooled OR of diabetes for total PCBs increased from 1.70 (95% CI: 1.28, 2.27) to 2.05 (95% CI: 1.41, 2.98) after results from the Yucheng cohort were excluded (Wang et al. 2008). Pooled data from our study and three previous investigations (Lee et al. 2011; Turyk et al. 2009; Vasiliu et al. 2006) did not support a nonlinear relationship between total PCB concentrations and diabetes (*p*_nonlinearity_ = 0.99) [see Supplemental Material, Figure S1 (http://dx.doi.org/10.1289/ehp.1205248)], although the power for detecting such a relationship was limited in the present analysis. Assuming a linear relationship, we estimated an OR of 1.06 (95% CI: 1.02, 1.09) per nanogram per gram serum weight increase in total PCBs. We could not examine dose–response relations for individual POPs because of insufficient data.

## Discussion

In this post hoc analysis using data from two prospective nested case–control studies among U.S. women, we found that plasma concentrations of some POPs, particularly HCB, were associated with increased risk of developing T2D. This observation was supported by a meta-analysis of our data pooled with six other prospective studies that demonstrated positive overall associations for HCB and total PCB concentrations with incident diabetes.

To our knowledge, this is the first prospective study to report a significant association between plasma HCB concentrations and risk of T2D. In a cross-sectional survey in a heavily polluted area of Eastern Slovakia, high HCB concentrations (> 1,364 vs. < 214 ng/g blood lipids) were significantly associated with prediabetes prevalence, but not with diabetes prevalence (Ukropec et al. 2010). In contrast, HCB was significantly associated with diabetes status in cross-sectional studies among Koreans (Son et al. 2010) and Native Americans (Mohawk) (Codru et al. 2007). After pooling our results with those from the Coronary Artery Risk Development in Young Adults (CARDIA) cohort (Lee et al. 2010) and the Prospective Investigation of the Vasculature in Uppsala Seniors study (Lee et al. 2011), we found a pooled RR of 2.0, comparing high with low HCB concentrations. The mechanisms that may underlie our observations are largely unknown. HCB is a toxic chemical that has a long elimination half-life. Therefore, it has been used as a “model chemical” to predict the ultimate fate of other POPs (Barber et al. 2005) and may only serve as a marker reflecting long-term exposure of mixed POPs. In our study and previous reports (Lee et al. 2006), HCB was positively correlated with most other POPs considered. Therefore, it is difficult to attribute effects to HCB alone. Previous studies also documented significant correlations between HCB and diabetes risk factors, including higher BMI (Lee et al. 2007a) and impaired fasting glucose (Langer et al. 2007), although plasma HCB was not significantly associated with BMI in our investigation in the NHS. More studies are needed to clarify potential mechanisms and establish the plausibility of the epidemiological associations.

In comparison to HCB, there is better evidence from animal experiments and human studies to support a causal role of PCBs and DDT in the etiology of T2D. An animal study showed that rats fed with fat rich in lipophilic POPs (primarily consisting of DDT, DDE, and PCBs) developed abdominal obesity and insulin insensitivity (Ruzzin et al. 2010). In addition, each doubling of PCB concentrations was associated with a 7% lower fasting insulin concentration in elderly Faroese residents with highly elevated PCB exposures from contaminated seafood (Grandjean et al. 2011). Moreover, high POP concentrations in the human body were associated with impaired glucose tolerance in Greenland Inuit who had much higher POP exposures in comparison with the general population (Jørgensen et al. 2008), as well as among the U.S. population with background POP exposure levels (Lee et al. 2007a). Animal studies have shown that some other polychlorinated compounds, such as 2,3,7,8-tetrachlorodibenzo-*p*-dioxin and 1,2,3,4,7,8-hexachlorodibenzo-*p*-dioxin, may impair glucose metabolism and regulation by reducing glucose uptake (Enan and Matsumura 1994) or by inhibiting the expression of insulin-like growth factor 1 and its binding protein (Croutch et al. 2005; Olsen et al. 1994). Despite this evidence, only a small number of prospective human studies have been conducted to investigate the association of PCBs, DDT, and DDE with diabetes. In a prospective study among Great Lakes sport fish consumers, higher DDE concentrations, but not total PCBs, were significantly associated with risk of diabetes (Turyk et al. 2009). Similarly, Rignell-Hydbom et al. (2009) found that DDE, but not PCB-153, showed a significant relationship with T2D in a case–control study among Swedish women, although the relationship was only evident among cases diagnosed ≥ 7 years after baseline. More recently, among a Swedish elderly population, Lee et al. (2011) documented that PCB-138, total PCBs, and summed values of DDE, *trans*-nonachlor, and HCB, were significantly associated with incident T2D. Inconsistencies between studies may be partially explained by differences in age, sex, or other characteristics and differences in POP exposure distributions among study populations. More important, most of these studies were based on small samples. In our meta-analysis, after we pooled all existing data, we observed a significant RR of 1.70 comparing high to low total PCB concentrations. All of these POPs have long elimination half-lives and they can still be detected among the U.S. population despite the fact that the use of these chemicals has been banned since the 1970s (Lee et al. 2010). Our findings suggest that past accumulation and continued exposure of these persistent pollutants may be a potent risk factor for developing diabetes.

The major strengths of our study included a prospective study design, a long follow-up of 18 years for the analysis of NHS data, and the use of meta-analysis to combine the NHS estimates with data from previous studies. There are several limitations of this study as well. First, we explored the association between POP concentrations and incident diabetes using existing data from two cancer case–control studies in the NHS. Because these studies were not designed to evaluate diabetes risk and because our participants were female registered nurses who were primarily white, results generated from these participants may have restricted generalizability, even within the entire NHS cohort. Further, we only accumulated a small number of diabetes cases from these two studies, resulting in limited statistical power. Second, the POP concentrations were measured by different methods in the two studies. However, we conducted study-specific analyses to account for between-study variation, and when we used a standardized score that minimized this between-study variation in a sensitivity analysis, we observed largely similar results. Third, the studies included in the meta-analysis measured various, intercorrelated individual POPs with different concentrations. We cannot exclude the possibility that the significant associations observed for HCB and total PCBs may be actually due to the effects of individual PCB congeners or other POPs. Fourth, we have limited statistical power for testing any nonlinear relationship between total PCBs and risk of diabetes in this meta-analysis. In addition, the dose–response relationship for individual POPs may be at least partly driven by individual or joint effects of POPs, which may vary depending on the concentrations of the individual POPs involved. Lee et al. (2010) found inverted U-shaped associations of diabetes with a summary measure of 31 POPs, and with certain individual PCB congeners, in the CARDIA study. Like that of other endocrine disruptors, the relationship between POPs and diabetes risk may depend on the level of exposure, i.e., POPs might increase diabetes risk monotonically at low dose, whereas at higher doses the effects of POPs may plateau or even decline (Daston et al. 2003; Welshons et al. 2003). Clearly, more evidence is needed to shed light on this complex dose–response relationship. Fifth, although tests of heterogeneity were far from significant, the power to detect heterogeneity was low, and the studies included in the meta-analysis differed with regard to age, sex, race, diabetes ascertainment or classification, and other characteristics that may modify the associations of interest. Sixth and last, although we observed significant associations for HCB and total PCBs with diabetes risk in the meta-analysis, we cannot rule out the possibility that these associations may have been due to other POPs, other unmeasured chemicals, residual confounding or other sources of bias, or chance. Moreover, the association between HCB and diabetes has been evaluated in only two prior prospective studies. More prospective studies with larger sample size and long follow-up duration are needed, and such studies should include HCB to confirm or refute the findings in this meta-analysis.

## Conclusions

Our estimates based on two prospective studies and a meta-analysis indicate that higher plasma HCB and total PCB concentrations are significantly associated with incident T2D. These findings support a positive association between POP exposure and risk of T2D.

## Supplemental Material

(365 KB) PDF
