# Supplementary material for: Persistent Organic Pollutants and Type 2 Diabetes: A Prospective Analysis in the Nurses’ Health Study and Meta-analysis
Source: Environ Health Perspect. 2012 Nov 5;121(2):153–61. doi: 10.1289/ehp.1205248 (PMC3569682; doi:10.1289/ehp.1205248)
Supplement: (365 KB) PDF [file ehp.1205248.s001.pdf]

Supplemental Material

Persistent Organic Pollutants and Type 2 Diabetes: A Prospective Analysis in the Nurses' Health Study and Meta-analysis

Hongyu Wu, Kimberly A. Bertrand, Anna L. Choi, Frank B. Hu, Francine Laden, Philippe Grandjean, Qi Sun

Table of Content

Search Terms for Meta-analysis……………………………………………………………………2

Table S1. Characteristics of study participants at baseline in 1990 by cancer status, the Nurses' Health Study……………………………………………………………………………..3

Table S2. Age-adjusted Spearman correlation coefficients between persistent organic pollutants among non-diabetic participants, the Nurses' Health Study………………………………….5

Table S3. Adjusted ORs (95% CIs) of incident diabetes per SD increment of natural log-transformed lipid-standardized plasma persistent organic pollutant concentrations, the Nurses' Health Study……………………………………………………………………………..6

Table S4. Adjusted ORs (95% CIs) of incident diabetes according to tertiles of plasma weight-adjusted plasma persistent organic pollutant concentrations (ng/g plasma weight), the Nurses' Health Study……………………………………………………………………………..8

Table S5. Pooled adjusted ORs (95% CIs) of incident diabetes according to tertiles of plasma weight-adjusted plasma persistent organic pollutant concentrations (ng/g plasma weight), the Nurses' Health Study………………………………………………………………………10

Table S6. Adjusted ORs (95% CIs) of incident diabetes according to tertiles of lipid-standardized plasma persistent organic pollutant concentrations (ng/g lipids), the Nurses' Health Study…12

Table S7. Adjusted ORs (95% CIs) of incident diabetes according to tertiles of lipid-standardized plasma persistent organic pollutant concentrations (ng/g lipids) among cancer free participants, the Nurses' Health Study…………………………………………………………………14

Figure S1. Dose-response relationship between concentrations of total PCBs and risk of type 2 diabetes in prospective studies…………………………………………………………………15

Search Terms for Meta-analysis

MEDLINE:

("Diabetes Mellitus"[Mesh] OR "diabetes"[All Fields]) AND ("Polychlorinated Biphenyls"[Mesh] OR "DDT"[Mesh] OR "Dichlorodiphenyl Dichloroethylene"[Mesh] OR "Hexachlorobenzene"[Mesh] OR "Hydrocarbons, Chlorinated"[Mesh] OR "Polychlorinated Biphenyls"[All Fields] OR “dichlorodiphenyltrichloroethane”[All Fields] OR "DDT"[All Fields] OR "Dichlorodiphenyl Dichloroethylene"[All Fields] OR "Hexachlorobenzene"[All Fields] OR “PCB”[All Fields] OR “DDE”[All Fields] OR “DDT”[All Fields] OR “HCB”[All Fields] OR “Pollutant”[All Fields] OR “Pesticide”[All Fields] OR “insecticide”[All Fields] OR “persistent organochlorine”[All Fields] OR “organochloride”[All Fields] OR “organochlorine”[All Fields] OR “chlorocarbon”[All Fields] OR “chlorinated hydrocarbon”[All Fields] OR “chlorinated solvent”[All Fields])

EMBASE:

“Diabetes” AND ("Polychlorinated Biphenyls" OR “dichlorodiphenyltrichloroethane” OR "DDT" OR "Dichlorodiphenyl Dichloroethylene" OR "Dichlorodiphenyldichloroethylene" OR "Hexachlorobenzene" OR “PCB” OR “DDE” OR “DDT” OR “HCB” OR “organochlorine Pesticide” OR “organochlorine insecticide” OR “persistent organochlorine Pollutant” OR “organochloride” OR “chlorocarbon” OR “chlorinated hydrocarbon” OR “chlorinated solvent”)

Table S1. Characteristics of study participants at baseline in 1990 by cancer status, the Nurses' Health Study. [a]

Variables	Breast Cancer study			Non-Hodgkin Lymphoma study		
	Cancer cases (n=334)	Cancer controls (n=339)	P value	Cancer cases (n=139)	Cancer controls (n=283)	*P* value
Age, years	58.7 ± 6.7	58.4 ± 6.8	0.50	58.7 ± 6.5	58.8 ± 6.5	0.86
BMI, kg/m^2	24.7 ± 4.1	25.0 ± 4.4	0.42	25.1 ± 4.1	25.0 ± 4.3	0.85
Smoking status, n (%)			0.57			0.64
Never smoked	147 (44.0)	161 (47.5)		59 (42.5)	132 (46.6)	
Past smoker	145 (43.4)	142 (41.9)		92 (44.6)	121 (42.8)	
Current smoker	42 (12.6)	36 (10.6)		18 (13.0)	30 (10.6)	
Alcohol drinking, n (%)			0.29			0.17
0g/day	62 (18.6)	59 (17.4)		29 (20.9)	83 (29.3)	
0.1-10g/day	176 (52.7)	198 (58.4)		78 (56.1)	145 (51.2)	
>10g/day	96 (28.7)	82 (24.2)		32 (23.0)	55 (19.4)	
Physical activity, MET-hours/week	16.3 ± 15.8	18.9 ± 17.2	0.04	16.5 ± 17.4	19.5 ± 38.9	0.28
Family history of diabetes, n (%)	80 (24.0)	82 (24.2)	0.94	39 (28.1)	83 (29.3)	0.79
Number of incident diabetes cases	3	21		2	22	
Number of deaths	75	51		63	38	
Total person-years	5288	5780		2012	4867	
Incident rate of type 2 diabetes, case/1,000 person-years	0.57	3.63	<0.001	0.99	4.52	0.005
PCB118, ng/g lipid	65.8 (45.8-86.2)	65.6 (47.2-85.6)	0.62	45.3 (34.1-67.0)	50.9 (34.4-78.2)	0.03
PCB138, ng/g lipid	94.6 (67.3-133.0)	94.2 (70.6-129.9)	0.80	64.0 (46.3-83.2)	63.4 (43.8-89.3)	0.19
PCB153, ng/g lipid	105.2 (80.2-143.6)	104.5 (80.9-140.7)	0.63	106.7 (79.5-130.8)	105.6 (79.0-141.0)	0.23
PCB180, ng/g lipid	76.4 (56.0-100.5)	73.3 (57.1-95.1)	0.87	74.7 (57.3-89.7)	70.3 (54.4-90.1)	0.41
∑PCBs (118,138,153,180), ng/g lipid	352.1 (262.7-459.1)	341.0 (265.9-448.8)	0.88	303.1 (227.3-354.4)	295.8 (217.8-395.2)	0.12
Total PCBs ng/g lipid [b]	768.2 (593.3-961.6)	724.0 (564.2-931.9)	0.71	622.3 (476.7-728.7)	618.9 (460.4-811.6)	0.50
p,p'-DDT, ng/g lipid	50.6 (33.3-92.4)	57.4 (32.2-96.9)	0.23	42.9 (27.7-69.3)	44.2 (28.2-67.2)	0.18

p,p'-DDE, ng/g lipid	734.8 (425.9-1209.6)	810.5 (487.9-1260.1)	0.02	945.1 (617.8-1879.9)	997.4 (556.2-1672.3)	0.83
HCB, ng/g lipid	28.9 (21.9-40.0)	30.4 (22.9-39.3)	0.69	35.5 (29.7-44.1)	37.2 (30.9-46.4)	0.50
Total plasma cholesterol, mg/dL	11.8 (8.0-16.1)	11.8 (8.5-16.6)	0.77	11.7 (7.8-17.7)	12.4 (8.0-19.0)	0.10
Plasma triglycerides, mg/dL	226.9 (197.0-253.0)	228.1 (203.0-253.1)	0.45	212.0 (186.0-240.0)	223.0 (191.0-246.0)	0.64

Abbreviations: PCB, polychlorinated biphenyls; DDT, *p, p'*-dichlorodiphenyltrichloroethane; DDE, *p, p'*-dichlorodiphenyldichloroethylene; HCB, hexachlorobenzene.

[a] Data are mean ± SD or median (inter-quartile range) for continuous variables or % for categorical variables, unless otherwise indicated. *P* values were calculated by *t* tests for continuous variables expressed as mean ± SD, Wilcoxon rank-sum tests for continuous variables expressed as median (inter-quartile range), and chi-square tests for categorical variables.

[b] Total PCBs were summed values of 22 PCB congeners in the breast cancer study and 56 PCB congeners in the non-Hodgkin lymphoma study.

Table S2. Age-adjusted Spearman correlation coefficients between persistent organic pollutants among non-diabetic participants, the Nurses' Health Study.

Breast Cancer study (n=649)	BMI	PCB118	PCB138	PCB153	PCB180	*p,p'*-DDT	*p,p'*-DDE
PCB118	-0.01 NS						
PCB138	-0.11	0.77					
PCB153	-0.21	0.80	0.89				
PCB180	-0.26	0.56	0.69	0.77			
p,p'-DDT	0.004 NS	0.30	0.24	0.26	0.21		
p,p'-DDE	0.03 NS	0.42	0.47	0.49	0.40	0.15	
HCB	-0.02 NS	0.42	0.36	0.44	0.45	0.15	0.45

Non-Hodgkin Lymphoma study (n=398)	BMI	PCB118	PCB138	PCB153	PCB180	*p,p'*-DDT	*p,p'*-DDE
PCB118	0.17						
PCB138	0.001 NS	0.79					
PCB153	-0.10 NS	0.76	0.95				
PCB180	-0.20	0.53	0.70	0.83			
p,p'-DDT	0.20	0.53	0.52	0.48	0.32		
p,p'-DDE	0.07 NS	0.47	0.52	0.47	0.33	0.63	
HCB	0.03 NS	0.42	0.38	0.42	0.46	0.40	0.49

NS, non-statistically significant. All correlation coefficients were adjusted for age at blood draw. *P*<0.05 unless indicated as NS.

Table S3. Adjusted ORs (95% CIs) of incident diabetes per SD increment of natural log-transformed lipid-standardized plasma persistent organic pollutant concentrations, the Nurses' Health Study. [a]

	Breast cancer study		Non-Hodgkin lymphoma study		Combined	
Ln (POP)	OR (95% CI)	*P*	OR (95% CI)	*P*	OR (95% CI)	*P*
Ln HCB						
Model 1 [b]	2.40 (0.85, 6.74)	0.10	2.53 (0.60, 10.7)	0.21	2.44 (1.05, 5.66)	0.04
Model 2 [c]	2.11 (0.77, 5.82)	0.15	3.05 (0.70, 13.3)	0.14	2.38 (1.03, 5.48)	0.04
Ln DDE						
Model 1 [b]	1.15 (0.65, 2.01)	0.64	0.95 (0.57, 1.61)	0.86	1.04 (0.71, 1.52)	0.85
Model 2 [c]	1.06 (0.60, 1.86)	0.84	1.01 (0.58, 1.76)	0.97	1.03 (0.70, 1.53)	0.87
Ln DDT						
Model 1 [b]	1.12 (0.65, 1.94)	0.68	1.15 (0.57, 2.30)	0.7	1.13 (0.74, 1.74)	0.57
Model 2 [c]	1.09 (0.63, 1.88)	0.75	1.27 (0.61, 2.62)	0.53	1.15 (0.74, 1.78)	0.53
Ln PCB118						
Model 1 [b]	1.87 (0.79, 4.40)	0.16	1.42 (0.66, 3.06)	0.37	1.60 (0.90, 2.84)	0.11
Model 2 [c]	1.57 (0.65, 3.78)	0.31	1.31 (0.61, 2.82)	0.49	1.42 (0.79, 2.53)	0.24
Ln PCB138						
Model 1 [b]	0.95 (0.39, 2.31)	0.91	0.86 (0.37, 2.00)	0.73	0.90 (0.49, 1.66)	0.74
Model 2 [c]	0.79 (0.33, 1.93)	0.61	0.90 (0.39, 2.10)	0.81	0.85 (0.46, 1.57)	0.60
Ln PCB153						
Model 1 [b]	2.85 (1.11, 7.33)	0.03	0.83 (0.29, 2.42)	0.73	1.66 (0.82, 3.37)	0.16
Model 2 [c]	2.37 (0.91, 6.21)	0.08	0.87 (0.30, 2.56)	0.8	1.52 (0.74, 3.12)	0.25
Ln PCB180						
Model 1 [b]	1.55 (0.58, 4.15)	0.38	0.75 (0.21, 2.65)	0.65	1.18 (0.54, 2.56)	0.68
Model 2 [c]	1.45 (0.53, 3.91)	0.47	0.84 (0.24, 2.92)	0.79	1.17 (0.54, 2.54)	0.69
Ln ∑ PCBs (118,138,153,180)						
Model 1 [b]	2.06 (0.76, 5.60)	0.16	0.95 (0.33, 2.74)	0.92	1.43 (0.69, 2.96)	0.34
Model 2 [c]	1.72 (0.62, 4.79)	0.30	0.96 (0.34, 2.77)	0.95	1.30 (0.62, 2.71)	0.49
Ln Total PCBs						
Model 1 [b]	1.83 (0.63, 5.33)	0.27	0.97 (0.30, 3.17)	0.96	1.37 (0.62, 3.04)	0.43
Model 2 [c]	1.52 (0.51, 4.50)	0.45	1.02 (0.32, 3.26)	0.98	1.26 (0.57, 2.79)	0.57

Ln Total POPs						
Model 1 [b]	1.36 (0.53, 3.52)	0.52	1.02 (0.46, 2.30)	0.96	1.25 (0.54, 2.90)	0.61
Model 2 [c]	1.62 (0.64, 4.13)	0.31	1.13 (0.49, 2.63)	0.78	1.55 (0.68, 3.54)	0.30

Abbreviations: PCB, polychlorinated biphenyls; DDT, *p, p'*-dichlorodiphenyltrichloroethane; DDE, *p, p'*-dichlorodiphenyldichloroethylene; HCB, hexachlorobenzene, POP, persistent organic pollutants.

[a] ORs (95% CIs) were estimated using logistic regression. The combined results were derived using a fixed-effects model. Total PCBs were summed values of 22 PCB congeners in the breast cancer study and 56 PCB congeners in the non-Hodgkin lymphoma study.

[b] Model 1: adjusted for age (years), smoking status (never/current smoker/ past smoker), alcohol intake (g/d: 0, 0.1-10 and >10), physical activity (MET-hours/week), family history of diabetes (yes/no), baseline BMI in 1990, total plasma cholesterol (mg/dL), and plasma triglyceride (mg/dL).

[c] Model 2: further adjusted for cancer case-control status.

Table S4. Adjusted ORs (95% CIs) of incident diabetes according to tertiles of plasma weight-adjusted plasma persistent organic pollutant concentrations (ng/g plasma weight), the Nurses' Health Study.[a]

POP	Breast cancer study Tertile 1	Breast cancer study Tertile 2	Breast cancer study Tertile 3	Breast cancer study *P* for trend	Non-Hodgkin lymphoma study Tertile 1	Non-Hodgkin lymphoma study Tertile 2	Non-Hodgkin lymphoma study Tertile 3	Non-Hodgkin lymphoma study *P* for trend
HCB [b]								
Median	0.15	0.24	0.35		0.20	0.28	0.38	
Case/control	6/330	8/161	10/158		5/206	10/96	9/96	
Model 1 [c]	1.00	3.60 (1.03, 12.6)	4.21 (1.15, 15.4)	0.03	1.00	3.91 (1.16, 13.2)	3.35 (0.91, 12.4)	0.07
Model 2 [d]	1.00	2.68 (0.75, 9.55)	3.98 (1.10, 14.5)	0.03	1.00	4.07 (1.17, 14.1)	3.75 (0.98, 14.5)	0.06
p,p'-DDE								
Median	2.35	5.42	10.37		2.84	6.84	14.70	
Case/control	8/216	6/219	10/214		5/135	6/135	13/128	
Model 1 [c]	1.00	0.74 (0.23, 2.37)	1.01 (0.33, 2.13)	0.92	1.00	0.79 (0.22, 2.86)	1.64 (0.51, 5.27)	0.25
Model 2 [d]	1.00	0.62 (0.18, 2.14)	0.91 (0.29, 2.92)	0.98	1.00	0.66 (0.18, 2.51)	1.69 (0.52, 5.49)	0.19
p,p'-DDT								
Median	0.19	0.38	0.81		0.15	0.30	0.58	
Case/control	8/216	4/221	12/212		4/136	7/134	13/128	
Model 1 [c]	1.00	0.39 (0.10, 1.46)	1.14 (0.41, 3.20)	0.47	1.00	1.11 (0.29, 4.26)	1.78 (0.50, 6.32)	0.29
Model 2 [d]	1.00	0.42 (0.11, 1.65)	1.01 (0.35, 2.90)	0.69	1.00	0.92 (0.23, 3.64)	1.64 (0.46, 5.84)	0.30
PCB118								
Median	0.28	0.46	0.73		0.20	0.34	0.61	
Case/control	4/220	7/218	13/211		4/136	7/134	13/128	
Model 1 [c]	1.00	2.16 (0.57, 8.11)	3.22 (0.87, 12.0)	0.09	1.00	1.47 (0.37, 5.82)	2.54 (0.65, 9.94)	0.15
Model 2 [d]	1.00	2.22 (0.58, 8.47)	2.75 (0.72, 10.5)	0.16	1.00	1.41 (0.34, 5.80)	2.32 (0.57, 9.50)	0.21
PCB138								
Median	0.42	0.66	1.05		0.26	0.42	0.70	
Case/control	5/219	9/216	10/214		4/136	11/130	9/132	
Model 1 [c]	1.00	1.44 (0.43, 4.87)	1.72 (0.49, 6.05)	0.42	1.00	2.01 (0.59, 6.93)	1.09 (0.28, 4.19)	0.74
Model 2 [d]	1.00	1.44 (0.42, 4.95)	1.60 (0.45, 5.68)	0.50	1.00	2.41 (0.68, 8.58)	1.07 (0.27, 4.23)	0.64
PCB153								
Median	0.48	0.75	1.13		0.47	0.73	1.07	
Case/control	5/219	9/215	10/215		6/134	9/132	9/132	

Model 1 [c]	1.00	1.92 (0.56, 6.55)	2.55 (0.68, 9.55)	0.18	1.00	1.24 (0.40, 3.87)	0.80 (0.23, 2.74)	0.63
Model 2 [d]	1.00	1.89 (0.54, 6.61)	2.31 (0.60, 8.88)	0.25	1.00	1.31 (0.42, 4.15)	0.72 (0.20, 2.55)	0.52
PCB180								
Median	0.35	0.52	0.76		0.34	0.49	0.69	
Case/control	7/217	9/216	8/216		9/131	5/136	10/131	
Model 1 [c]	1.00	1.36 (0.44, 4.20)	1.07 (0.28, 4.07)	0.94	1.00	0.46 (0.13, 1.62)	0.78 (0.23, 2.70)	0.86
Model 2 [d]	1.00	1.33 (0.42, 4.26)	1.02 (0.25, 4.15)	0.98	1.00	0.44 (0.12, 1.61)	0.84 (0.23, 3.06)	0.97
Σ PCBs (118, 138, 153, 180)								
Median	1.57	2.41	3.63		1.35	2.00	2.99	
Case/control	6/218	8/217	10/214		4/136	11/130	9/132	
Model 1 [c]	1.00	1.32 (0.40, 4.39)	1.48 (0.42, 5.29)	0.57	1.00	2.02 (0.58, 6.98)	1.20 (0.30, 4.72)	0.91
Model 2 [d]	1.00	1.31 (0.38, 4.47)	1.41 (0.39, 5.14)	0.63	1.00	2.32 (0.65, 8.29)	1.15 (0.28, 4.70)	0.79
Total PCBs								
Median	3.52	5.17	7.64		2.86	4.24	6.18	
Case/control	4/220	12/213	8/216		4/136	9/132	11/130	
Model 1 [c]	1.00	3.64 (1.02, 13.0)	1.53 (0.36, 6.43)	0.91	1.00	1.65 (0.45, 6.02)	1.54 (0.38, 6.23)	0.65
Model 2 [d]	1.00	4.06 (1.08, 15.3)	1.53 (0.35, 6.58)	0.93	1.00	1.78 (0.48, 6.68)	1.44 (0.35, 6.01)	0.78
Total POPs								
Median	7.19	11.8	18.5		6.75	12.2	21.5	
Case/control	6/218	7/218	11/213		5/135	8/133	11/130	
Model 1 [c]	1.00	1.15 (0.34, 3.88)	1.53 (0.46, 5.16)	0.47	1.00	1.02 (0.30, 3.48)	1.23 (0.36, 4.19)	0.71
Model 2 [d]	1.00	1.00 (0.29, 3.45)	1.22 (0.35, 4.27)	0.73	1.00	0.88 (0.25, 3.13)	1.31 (0.38, 4.56)	0.56

[a] ORs (95% CIs) were estimated using logistic regression. Total PCBs were summed values of 22 PCB congeners in the breast cancer study and 56 PCB congeners in the non-Hodgkin lymphoma study.

[b] In order to include T2D cases in the lowest tertile of HCB, participants were categorized using the following cutpoints: ≤ median, median to 75 percentile, and ≥75 percentile.

[c] Model 1: adjusted for age (years), smoking status (never/current smoker/ past smoker), alcohol intake (g/d: 0, 0.1-10 and >10), physical activity (MET-hours/week), family history of diabetes (yes/no), baseline BMI in 1990, total plasma cholesterol (mg/dL), and plasma triglycerides (mg/dL).

[d] Model 2: further adjusted for cancer case-control status.

Table S5. Pooled adjusted ORs (95% CIs) of incident diabetes according to tertiles of plasma weight-adjusted plasma persistent organic pollutant concentrations (ng/g plasma weight), the Nurses' Health Study. [a]

	Combined			
POP	Tertile 1	Tertile 2	Tertile 3	*P* for trend
HCB [b]				
Case/control	11/536	18/257	19/254	
Model 1 [c]	1.00	3.76 (1.57, 9.00)	3.76 (1.50, 9.44)	0.005
Model 2 [d]	1.00	3.32 (1.36, 8.07)	3.87 (1.52, 9.83)	0.004
p,p'-DDE				
Case/control	13/351	12/354	23/342	
Model 1 [c]	1.00	0.76 (0.32, 1.82)	1.25 (0.55, 2.81)	0.33
Model 2 [d]	1.00	0.63 (0.26, 1.57)	1.24 (0.54, 2.83)	0.27
p,p'-DDT				
Case/control	12/352	11/355	25/340	
Model 1 [c]	1.00	0.64 (0.25, 1.65)	1.31 (0.59, 2.91)	0.28
Model 2 [d]	1.00	0.62 (0.23, 1.63)	1.21 (0.54, 2.74)	0.39
PCB118				
Case/control	8/356	14/352	26/339	
Model 1 [c]	1.00	1.76 (0.68, 4.56)	2.77 (1.08, 7.12)	0.03
Model 2 [d]	1.00	1.78 (0.67, 4.70)	2.52 (0.95, 6.67)	0.06
PCB138				
Case/control	9/355	20/346	19/346	
Model 1 [c]	1.00	1.69 (0.71, 4.03)	1.37 (0.55, 3.43)	0.66
Model 2 [d]	1.00	1.87 (0.77, 4.54)	1.35 (0.53, 3.42)	0.75
PCB153				
Case/control	11/353	18/347	19/347	
Model 1 [c]	1.00	1.51 (0.66, 3.48)	1.35 (0.54, 3.33)	0.53
Model 2 [d]	1.00	1.56 (0.67, 3.64)	1.26 (0.50, 3.18)	0.65
PCB180				
Case/control	16/348	14/352	18/347	
Model 1 [c]	1.00	0.85 (0.37, 1.97)	0.89 (0.36, 2.22)	0.93
Model 2 [d]	1.00	0.83 (0.35, 1.96)	0.94 (0.36, 2.43)	0.97
Σ PCBs (118, 138, 153, 180)				
Case/control	10/354	19/347	19/347	
Model 1 [c]	1.00	1.62 (0.68, 3.82)	1.31 (0.51, 3.33)	0.75
Model 2 [d]	1.00	1.76 (0.73, 4.26)	1.29 (0.50, 3.37)	0.83
Total PCBs				
Case/control	8/356	21/345	19/346	
Model 1 [c]	1.00	2.37 (0.96, 5.83)	1.47 (0.54, 4.00)	0.77
Model 2 [d]	1.00	2.60 (1.03, 6.57)	1.45 (0.52, 4.03)	0.82
Total POPs				
Case/control	11/353	15/351	22/343	
Model 1 [c]	1.00	1.08 (0.45, 2.57)	1.36 (0.58, 3.22)	0.47
Model 2 [d]	1.00	0.94 (0.39, 2.29)	1.29 (0.54, 3.11)	0.47

[a] ORs (95% CIs) were estimated using logistic regression. The combined results were derived using a fixed-effects model. Total PCBs were summed values of 22 PCB congeners in the breast cancer study and 56 PCB congeners in the non-Hodgkin lymphoma study.

[b] In order to include T2D cases in the lowest tertile of HCB, participants were categorized using the following cutpoints: ≤ median, median to 75 percentile, and ≥75 percentile.

[c] Model 1: adjusted for age (years), smoking status (never/current smoker/ past smoker), alcohol intake (g/d: 0, 0.1-10 and >10), physical activity (MET-hours/week), family history of diabetes (yes/no), baseline BMI in 1990, total plasma cholesterol (mg/dL), and plasma triglycerides (mg/dL).

[d] Model 2: further adjusted for cancer case-control status.

Table S6. Adjusted ORs (95% CIs) of incident diabetes according to tertiles of lipid-standardized plasma persistent organic pollutant concentrations (ng/g lipids), the Nurses' Health Study.[a]

	Pooled datasets of breast cancer study and non-Hodgkin lymphoma study			
POP	Tertile 1	Tertile 2	Tertile 3	*P* for trend
HCB				
Case/control	8/349	17/349	23/349	
Model 1 [b]	1.00	2.14 (0.86, 5.32)	3.79 (1.54, 9.34)	0.003
Model 2 [c]	1.00	1.89 (0.75, 4.79)	3.35 (1.34, 8.37)	0.007
p,p'-DDE				
Case/control	11/349	19/349	18/349	
Model 1 [b]	1.00	1.67 (0.76, 3.65)	1.47 (0.65, 3.29)	0.39
Model 2 [c]	1.00	1.53 (0.68, 3.43)	1.51 (0.66, 3.44)	0.35
p,p'-DDT				
Case/control	14/349	15/349	19/349	
Model 1 [b]	1.00	0.92 (0.42, 1.98)	1.03 (0.49, 2.18)	0.93
Model 2 [c]	1.00	0.98 (0.45, 2.16)	1.06 (0.49, 2.27)	0.88
PCB118				
Case/control	11/349	17/349	20/349	
Model 1 [b]	1.00	1.52 (0.67, 3.45)	1.66 (0.73, 3.77)	0.24
Model 2 [c]	1.00	1.50 (0.65, 3.47)	1.58 (0.68, 3.64)	0.31
PCB138				
Case/control	15/349	17/349	16/349	
Model 1 [b]	1.00	1.06 (0.51, 2.23)	1.12 (0.53, 2.40)	0.76
Model 2 [c]	1.00	1.04 (0.49, 2.23)	1.07 (0.49, 2.34)	0.86
PCB153				
Case/control	15/349	17/349	16/349	
Model 1 [b]	1.00	1.13 (0.53, 2.41)	1.44 (0.66, 3.15)	0.37
Model 2 [c]	1.00	1.16 (0.54, 2.53)	1.39 (0.62, 3.11)	0.42
PCB180				
Case/control	17/349	19/349	12/349	
Model 1 [b]	1.00	1.49 (0.72, 3.07)	1.23 (0.53, 2.86)	0.56
Model 2 [c]	1.00	1.46 (0.69, 3.08)	1.33 (0.56, 3.17)	0.48
∑ PCBs (118,138,153,180)				
Case/control	15/349	17/349	16/349	
Model 1 [b]	1.00	1.13 (0.54, 2.39)	1.20 (0.55, 2.59)	0.65
Model 2 [c]	1.00	1.14 (0.53, 2.45)	1.19 (0.54, 2.63)	0.66
Total PCBs				
Case/control	15/349	17/349	16/349	
Model 1 [b]	1.00	1.23 (0.58, 2.60)	1.24 (0.57, 2.70)	0.58
Model 2 [c]	1.00	1.41 (0.65, 3.04)	1.24 (0.56, 2.76)	0.59
Total POPs				
Case/control	11/349	16/349	21/349	
Model 1 [b]	1.00	1.55 (0.69, 3.49)	2.01 (0.91, 4.44)	0.09
Model 2 [c]	1.00	1.39 (0.62, 3.19)	1.86 (0.83, 4.17)	0.13

[a] ORs (95% CIs) were estimated using logistic regression. Before pooling individual-level data of the non-Hodgkin lymphoma and breast cancer studies, within each dataset, a standardized score of each persistent organic pollutant was computed by using Rosner's non-parametric approach, the derived scores were then used to represent its distribution. Total PCBs were summed values of 22 PCB congeners in the breast cancer study and 56 PCB congeners in the non-Hodgkin lymphoma study.
[b] Model 1: adjusted for age (years), smoking status (never/current smoker/ past smoker), alcohol intake (g/d: 0, 0.1-10 and >10), physical activity (MET-hours/week), family history of diabetes (yes/no), baseline BMI in 1990, and data source (breast cancer study/ non-Hodgkin lymphoma study).
[c] Model 2: further adjusted for cancer case-control status.

Table S7. Adjusted ORs (95% CIs) of incident diabetes according to tertiles of lipid-standardized plasma persistent organic pollutant concentrations (ng/g lipids) among cancer free participants, the Nurses' Health Study. [a]

	Combined datasets of breast cancer study and non-Hodgkin lymphoma study			
POP	Tertile 1	Tertile 2	Tertile 3	*P* for trend
HCB				
Case/control	10/193	13/193	20/193	
Model 1 [b]	1.00	1.10 (0.43, 2.80)	2.47 (1.02, 5.99)	0.03
DDE				
Case/control	9/193	17/193	17/193	
Model 1 [b]	1.00	1.78 (0.74, 4.30)	1.85 (0.77, 4.49)	0.19
DDT				
Case/control	12/193	14/193	17/193	
Model 1 [b]	1.00	1.07 (0.46, 2.46)	1.15 (0.51, 2.59)	0.74
PCB118				
Case/control	10/193	15/193	18/193	
Model 1 [b]	1.00	1.35 (0.56, 3.26)	1.46 (0.61, 3.53)	0.41
PCB138				
Case/control	15/193	15/193	14/193	
Model 1 [b]	1.00	0.87 (0.39, 1.95)	0.93 (0.41, 2.09)	0.85
PCB153				
Case/control	14/193	15/193	14/193	
Model 1 [b]	1.00	1.04 (0.46, 2.36)	1.28 (0.54, 3.01)	0.58
PCB180				
Case/control	16/193	14/193	13/193	
Model 1 [b]	1.00	1.20 (0.53, 2.73)	1.41 (0.58, 3.44)	0.45
∑ PCBs (118,138,153,180)				
Case/control	14/193	15/193	14/193	
Model 1 [b]	1.00	1.05 (0.47, 2.35)	1.06 (0.46, 2.48)	0.89
Total PCBs				
Case/control	12/193	18/193	13/193	
Model 1 [b]	1.00	1.66 (0.73, 3.76)	1.20 (0.49, 2.92)	0.74
Total POPs				
Case/control	9/193	16/193	18/193	
Model 1 [b]	1.00	1.88 (0.77, 4.58)	2.12 (0.87, 5.13)	0.11

[a] ORs (95% CIs) were estimated using logistic regression. Before combining individual-level data from the non-Hodgkin lymphoma and breast cancer studies, within each dataset, a standardized score of each persistent organic pollutant was computed by using Rosner's non-parametric approach. The derived scores were then used to represent its distribution. Total PCBs were summed values of 22 PCB congeners in the breast cancer study and 56 PCB congeners in the non-Hodgkin lymphoma study.

[b]Model 1: adjusted for age (years), smoking status (never/current smoker/ past smoker), alcohol intake (g/d: 0, 0.1-10 and >10), physical activity (MET-hours/week), family history of diabetes (yes/no), and baseline BMI in 1990.

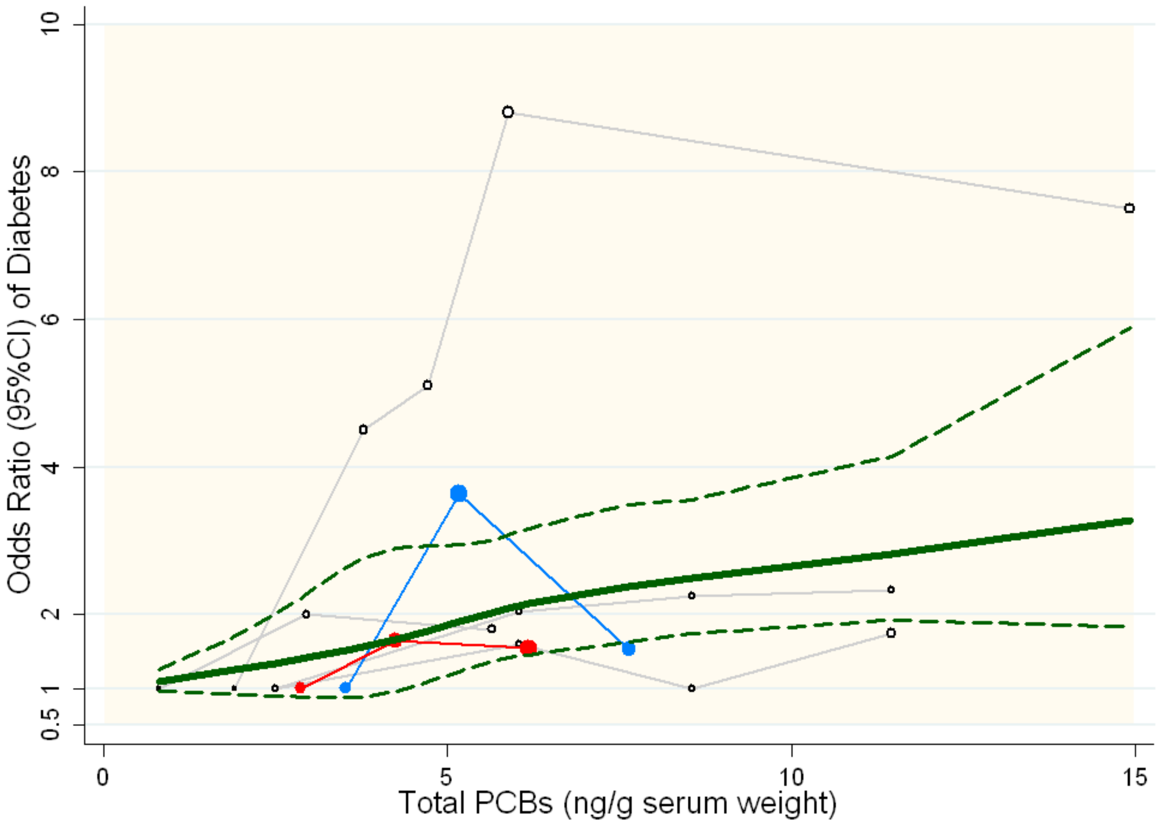

Figure S1. Dose-response relationship between concentrations of total PCBs and risk of type 2 diabetes in prospective studies. Solid line represents point estimates of association between total PCBs and diabetes risk, derived by using generalized least-squares method for trend estimation of summarized dose-response data; dashed lines are 95% CI of the point estimates. Filled blue circles and red circles are odds ratios corresponding to comparison categories in the breast cancer study and non-Hodgkin lymphoma study, respectively. The open circles are odds ratios from other contributing prospective studies (grey lines from top to bottom are Lee et al. 2011; Turyk et al. 2009; men and women in Vasiliu et al. 2006). Yucheng cohort (Wang et al. 2008) was excluded from this the dose-response analysis because this study included subjects who were poisoned by exposure to extraordinarily-high levels of PCBs and polychlorinated dibenzofurans (PCDFs) in contaminated cooking oil.
